# Complex B_1_^+^ mapping with Carr‐Purcell spin echoes and its application to electrical properties tomography

**DOI:** 10.1002/mrm.29020

**Published:** 2021-11-09

**Authors:** Santhosh Iyyakkunnel, Matthias Weigel, Carl Ganter, Oliver Bieri

**Affiliations:** ^1^ Division of Radiological Physics Department of Radiology University Hospital Basel Basel Switzerland; ^2^ Department of Biomedical Engineering University of Basel Basel Switzerland; ^3^ Translational Imaging in Neurology (ThINk) Basel Department of Biomedical Engineering Faculty of Medicine University Hospital Basel and University of Basel Basel Switzerland; ^4^ Neurologic Clinic and Policlinic, MS Center and Research Center for Clinical Neuroimmunology and Neuroscience Basel (RC2NB) University Hospital Basel and University of Basel Basel Switzerland; ^5^ Department of Radiology Klinikum rechts der Isar Technical University of Munich Munich Germany

**Keywords:** B_1_ mapping, CP, CPMG, electrical properties tomography, spin echoes

## Abstract

**Purpose:**

To present a new complex‐valued B_1_^+^ mapping method for electrical properties tomography using Carr‐Purcell spin echoes.

**Methods:**

A Carr‐Purcell (CP) echo train generates pronounced flip‐angle dependent oscillations that can be used to estimate the magnitude of B_1_^+^. To this end, a dictionary is used that takes into account the slice profile as well as T_2_ relaxation along the echo train. For validation, the retrieved B_1_^+^ map is compared with the actual flip angle imaging (AFI) method in a phantom (79 ε_0_, 0.34 S/m). Moreover, the phase of the first echo reflects the transceive phase. Overall, the CP echo train yields an estimate of the complex‐valued B_1_^+^, allowing electrical properties tomography with both permittivity and conductivity. The presented method is evaluated in phantom scans as well as for in vivo brain at 3 T.

**Results:**

In the phantom, the obtained magnitude B_1_^+^ maps retrieved from the CP echo train and the AFI method show excellent agreement, and both the reconstructed estimated permittivity (79 ± 3) ε_0_ and conductivity (0.35 ± 0.04) S/m values are in accordance with expectations. In the brain, the obtained electrical properties are also close to expectations. In addition to the retrieved complex B_1_^+^ information, the decay of the CP echo trains also yields an estimate for T_2_.

**Conclusion:**

The CP sequence can be used to simultaneously provide both B_1_^+^ magnitude and phase estimations, and therefore allows for full reconstruction of the electrical properties.

## 1 INTRODUCTION

During an MRI scan, the electromagnetic fields, such as the RF fields used for excitation, are affected by the tissues’ electrical properties (EPs), which in turn are sensitive to pathological changes and therefore show potential in diagnosis and treatment of cancer patients.^1^, ^2^ Getting the EPs from a conventional MRI scan was first mentioned by Haacke et al.^3^ in 1991, but this idea was not widely pursued because of “spurious phase effects” until after the turn of the century, when this technique would be first known as electric properties tomography (EPT).^4^, ^5^, ^6^

EPT exploits the relation between the EPs and the electromagnetic fields derived from the Maxwell equations. In fact, EPs depend on the curvature (second‐order derivative) of the complex RF transmit field (B_1_^+^) inside the scanned object, and thus require acquisitions with high SNR. Even though methods to estimate the complex‐valued B_1_^+^ have been reported,^7^, ^8^, ^9^ generally two separate scans are required: one to determine its amplitude and another to determine its phase. For the latter, a spin‐echo sequence is often used, as it combines high signal with robustness against field inhomogeneities. To measure the B_1_^+^ amplitude, one generally reverts to existing B_1_ mapping methods.^9^, ^10^, ^11^ These methods are based primarily on gradient‐echo sequences, as most applications for B_1_ mapping value speed over resolution. In the context of this work, a method termed B_1_‐TRAP (B_1_ mapping with the transient phase of unbalanced SSFP)^12^ stands out. This method retrieves the local B_1_ field from the oscillations of the transient phase of a coherent steady state sequence. It can thus be expected that a train of spin echoes should exhibit similar signal oscillations as a function of the flip angle. In fact, this flip angle–dependent oscillatory behavior for multi‐spin‐echo sequences, such as the ones suggested by Carr and Purcell (CP)^13^ or Meiboom and Gill (CPMG),^14^ has already been demonstrated.^15^, ^16^, ^17^

In this work, we will show that the local B_1_^+^ magnitude can be retrieved from a CP echo train in conjunction with an estimate for the transceive phase. As a result, a CP sequence offers a unique setting for EPT. Moreover, the enveloping decay of the CP echoes offers an estimate for the transverse relaxation time T_2_.

## 2 METHODS

**FIGURE 1:**
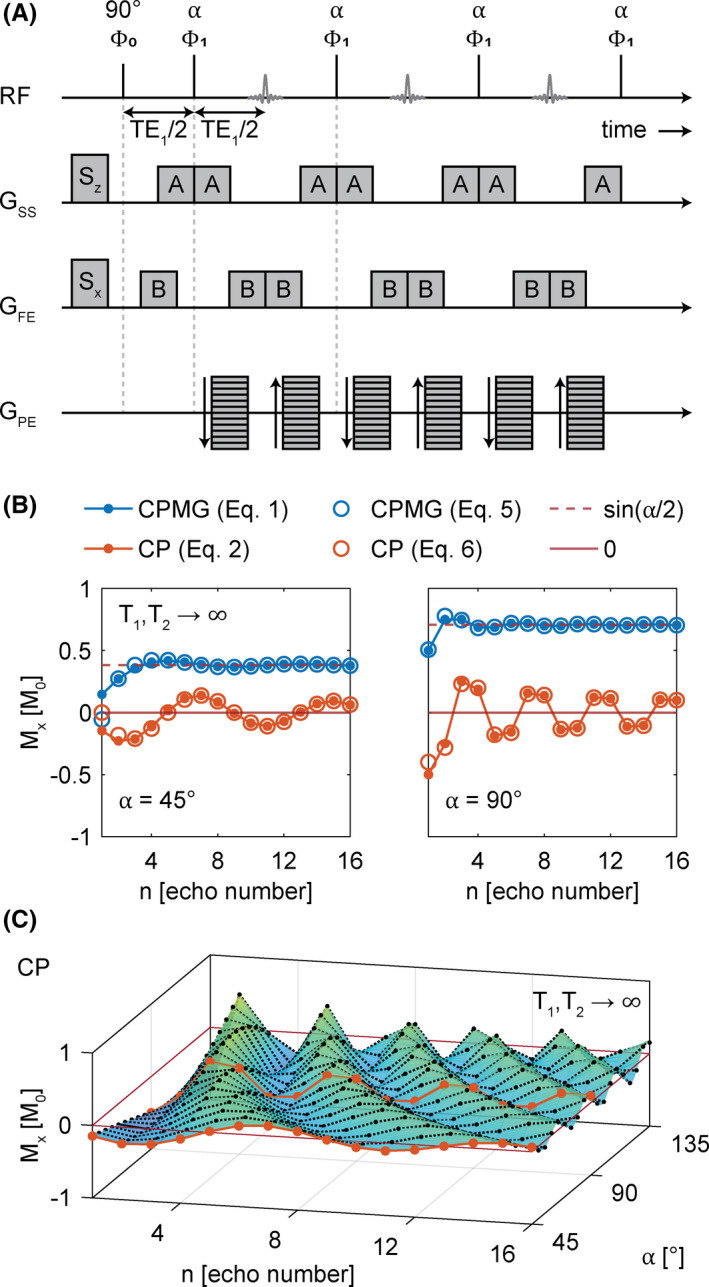
A, General simplified schematic of the multi‐spin‐echo sequence with equidistant echoes. B, Comparison between simulated values of echo amplitudes using analytic integral expressions from Equations 1 and 2 to compute the echo amplitudes (filled circles) and the analytic expressions from Equations 5 and 6, which are valid for large *n* (open circles). Both approaches do not consider relaxation effects. Echo amplitudes are shown for flip angles α = 45° (left) and α = 90° (right). C, Surface plot showing the flip‐angle dependency of the Carr‐Purcell (CP) multi‐echo signal. Abbreviation: CPMG, Carr‐Purcell‐Meiboom‐Gill

A multi‐spin‐echo sequence with equidistant echo spacing is shown in Figure 1A, where α denotes the flip angle of the refocusing pulse; Φ stands for the RF phase; and TE denotes the echo time (also indicating the temporal spacing between the application of RF pulses). In the CP scheme, all excitations are applied along the same axis; thus, $\Phi_{0}=\Phi_{1}$ applies.^13^ In the CPMG scheme, excitation and refocusing pulses are perpendicular to each other, and $\left|\Phi_{0}‐\Phi_{1}\right|=90^{\circ }$ applies.^14^ For the experiments described in the following, a prototype of such a multi‐spin‐echo sequence was used. By changing the RF phase of the excitation, the sequence was run in either CP or CPMG mode.

### 2.1 B_1_^+^ mapping with multi‐spin‐echo sequences

The echo amplitudes for both the CP and CPMG sequence as a function of the flip angle has been studied in the past.^15^, ^16^, ^17^, ^18^, ^19^ Zur^20^ derived analytic integral expressions for the *n* th echo intensity of CP, as well as CPMG, signals in the absence of relaxation from a vector model for the sequence’s pseudo steady state:

(1) $$I_{\mathrm{CPMG}}\left(n\right)=M_{0}\left(\sin\frac{\alpha }{2}+\frac{1}{\pi }\int_{0}^{\pi }\frac{\lambda^{2}}{1+\lambda^{2}}e^{in\psi }d\phi \right)$$

(2) $$I_{\mathrm{CP}}\left(n\right)=M_{0}\left(\frac{1}{\pi }\int_{0}^{\pi }e^{in\psi }d\phi \right)$$

with

(3) $$\psi ={2\;\cos}^{‐1}\left[\cos\frac{\alpha }{2}\cos\phi \right]$$

and

(4) $$\lambda =\frac{\cos\frac{\alpha }{2}\sin\phi }{\sin\frac{\alpha }{2}},$$

where 2ϕ is the phase accumulation of an isochromat during one TR, and $M_{0}$ is the equilibrium magnetization.

Despite the compact forms of Equations 1 and 2, it is not obvious how the B_1_^+^ amplitude can be retrieved from the echo amplitudes of CP and CPMG echo trains. Using a generating functions formalism, Lukzen et al^16^, ^17^ showed that the asymptotic behavior (n→∞) of Equations 1 and 2 is of the following form:

(5) $$I_{\mathrm{CPMG}}\left(n\to \infty \right)=M_{0}\left(\sin\frac{\alpha }{2}‐\frac{1}{2}\frac{1}{\sqrt{\pi \tan\left(\frac{\alpha }{2}\right)}}\frac{\cos\left(n\alpha ‐\frac{\pi }{4}\right)}{\sqrt{n^{3}}}\right)$$

(6) $$I_{\mathrm{CP}}\left(n\to \infty \right)=M_{0}\left(\tan\left(\frac{\alpha }{2}\right)\frac{1}{\sqrt{\pi \tan\left(\frac{\alpha }{2}\right)}}\frac{\cos\left(n\alpha +\frac{\pi }{4}\right)}{\sqrt{n}}\right)$$

Notably, these expressions converge to Zur’s^20^ exact solutions (Equations 1 and 2) already for n≳8. Equations 5 and 6 now clearly show that the echo amplitudes oscillate with a frequency of α/2π. This is illustrated in Figure 1B, where the echo intensities are plotted for both CP and CPMG, using Equations 1, 2, 5, and 6 and for flip angles $\alpha =45^{\circ }$ (Figure 1B, left) and $\alpha =90^{\circ }$ (Figure 1B, right). Figure 1B further confirms that the integral and explicit expressions for the echo amplitudes are identical for larger *n*. From this, a voxel‐wise analysis of the oscillatory behavior of the multi‐spin‐echo train can be used to yield an estimate for the local flip angle, which in turn can be used as a direct measure for the relative B_1_^+^ amplitude.^21^, ^22^ For convenience, the B_1_^+^ amplitude as a relative quantity will be referred to as just B_1_ in the following.

Even though CPMG and CP signals oscillate with the same flip angle–dependent frequency, they differ in other aspects, such as amplitude, asymptotic limit, and convergence speed (see Figure 1B). The amplitude of the damped oscillations in the two modes differs by a factor $2\tan\left(\alpha /2\right)$. Thus, for $\alpha =90^{\circ }$, the oscillatory amplitude is twice as high in CP as compared with CPMG. Conversely, in CPMG, a high signal intensity is maintained even for late echoes due to constructive interference of spin echoes and stimulated echoes and convergence to the asymptotic limit of $M_{0}\sin\left(\alpha /2\right)$.^14^, ^18^ In the CP sequence, destructive interference causes the signal to decay to zero, notably even in the absence of relaxation. Interestingly, from Equations 5 and 6, we see that CP signals converge with $1/\sqrt{n}$, whereas CPMG signals converge with ${\left(1/\sqrt{n}\right)}^{3}$. This results in a slower signal decay in CP than in CPMG (see Figure 1B), offering longer oscillatory traces. In summary, the CP sequence is much better suited for B_1_ mapping than CPMG and is thus used in the following.

### 2.2 Choice of the refocusing flip angle

The refocusing flip angle will determine the frequency of the measured signal (Equations 5 and 6). Based on our experience, the observed flip angles deviate approximately by about a factor of 0.5–1.3 from the nominal flip angle at 3 T. Thus, the choice of the nominal refocusing flip angle can be viewed as a trade‐off between high signal intensity, resulting RF power deposition (leading to the specific absorption rate) and the sampling of the oscillation frequency across the object. At high refocusing flip angles of α > 150°, specific absorption rate limits might be reached at 3 T, but the oscillation frequency also becomes aliased ($B_{1}∙\alpha$ > 180°). Thus, a refocusing flip angle of 90° appears to be a good compromise for the purpose of B_1_ mapping and is chosen for the following experiments.

### 2.3 Transmit phase estimation

The phase of a spin echo is free from external disturbances such as field inhomogeneities. Consequently, with the first echo, the phase directly at the time of excitation will be restored. This phase corresponds to the so‐called transceive phase, as it contains contributions from both the transmit field and the receive field or sensitivity.^23^, ^24^ For EPT, ideally the complex B_1_^+^ (i.e., the local B_1_^+^ magnitude and the transmit phase) enters the reconstruction. To get an estimate of the transmit phase, the so‐called transceive phase assumption is frequently used.^23^ The transceive phase assumption states that transmit and receive phases are almost equal under the conditions that the field strength is no greater than 3 T, the scanned object is of approximately cylindrical form, and a quadrature birdcage coil is used for excitation and reception.^23^ In the clinical setting, however, typically only multichannel receive coils are available, and proper estimation of the transceive phase depends on the coil combine method used.^25^, ^26^

Because the transceive phase is contained in the measurement, the uncertainty in the transceive map can be estimated by the inverse of the SNR of the spin‐echo magnitude image.^27^, ^28^ The magnitude SNR was obtained by the ratio of the average signal magnitude within the object and the SD of the real part of the image in the background (air).

### 2.4 Simulations and dictionary generation

In this study, a dictionary is used to retrieve the frequency of the oscillating echo intensities. Although the frequency of the acquired signals depends on the local B_1_^+^ magnitude, the signal decay is governed primarily by the transverse relaxation time^18^ and can be used to yield an estimate for T_2_.

For the generation of the dictionary, the CP pulse sequence was first simulated to yield the exact timing, shape, and amplitude of all RF and gradient pulses. Then, the tissue‐specific signal response was simulated with *MATLAB* (The MathWorks, Natick, MA) using the configuration model toolkit (CoMoTk)^29^ for a range of nominal flip angles and T_2_ values (taking into account slice profile effects). The slice profile was taken into account by simulating the effect of the RF pulses using the hard pulse approximation.^30^, ^31^ T_1_ was fixed at 1 second, as simulations showed negligible impact of common brain‐tissue T_1_ values on the signal. For T_2_, 21 logarithmically spaced points between 10 and 1000 ms were cubically interpolated to have a total of 41 points in this range. Then, the range of T_2_ was extended by 10 linearly spaced points between 1200 and 3000 ms. The relative B_1_ was varied linearly between 0.5 and 1.3 with steps of 0.01. Finally, the simulated CP signals were multiplied with $\sqrt{n}$ to compensate for the inherent CP decay (see Equation 6). Finding the best match of the measurement in the dictionary was performed by searching for the maximum Pearson correlation coefficient. To this end, the normalized measured complex‐valued signal was multiplied with all the normalized simulated signals from the dictionary. Normalization was performed by subtracting the signal’s mean and dividing by its SD. Because the input was partly complex‐valued, the resulting Pearson correlation coefficients were also complex‐valued, and the maximum was determined based on its magnitude.

Simulations and calculations were performed in *MATLAB* (R2019a) unless stated otherwise.

### 2.5 Imaging experiments

Imaging was performed in a phantom and for in vivo brain at 3 T (Magnetom Prisma; Siemens Healthcare, Erlangen, Germany) using the body coil for transmission and the manufacturer’s 20‐channel head and neck coil for reception. Phase images from multichannel coil data were reconstructed using the manufacturer’s “adaptive coil combine” method. To evaluate the appropriateness of the multichannel coil combination method for EPT, phantom scans were also performed with a 1H/23Na transmit/receive birdcage coil (RAPID Biomedical, Rimpar, Germany). If not otherwise explicitly stated, scanning was performed with the multichannel head and neck coil. In vivo human MR scans were approved by the local ethics committee, and informed written consent was obtained before the human experiments.

For all experiments, a 2D CP multi‐spin‐echo prototype sequence was used with spoiler and crusher gradient moments along the slice selection direction (S_z_ = 43.98 mT/m·ms and A = 24.75 mT/m·ms), as well as along the frequency encoding direction (S_x_ = 17.89 mT/m·ms and B = 46.17 mT/m·ms) (see Figure 1A). Within a TR of 2800 ms, 10 echoes were acquired with an echo spacing of 11 ms, starting at TE_1_ = 11 ms. To accelerate the scan the partially parallel acquisition technique, GRAPPA^32^ was used. To minimize crosstalk between excited slices, a total of 36 slices with 3‐mm slice thickness were split in two subsequently measured interleaved slice groups with a 3‐mm gap between slices. In plane, cartesian *k*‐space sampling was used for a 144 × 120 matrix and a 1.5 × 1.5 mm^2^ resolution. For EP reconstruction, the slices were interpolated to obtain an isotropic voxel size of 1.5 × 1.5 × 1.5 mm^3^. For excitation, a sinc‐shaped RF pulse with a time‐bandwidth product of 2 and thickness factor of 0.8 was used, while the refocusing pulses were sinc‐shaped pulses with a time‐bandwidth product of 4 and a thickness factor of 1.6. Both the excitation and refocusing pulses had a duration of 2.8 ms, and a Hanning filter was used to mitigate pulse truncation effects. The receiver bandwidth was set to 205 Hz/px, and acquisition was completed in less than 7 minutes.

For the phantom scans, a water bottle with a salt concentration of 2 g/L was used. The saline phantom had an estimated conductivity of 0.34 S/m and a permittivity of 79 ε_0_ at room temperature.^33^, ^34^ Tissue‐comparable relaxation times of approximately T_1_ ∼ 870 ms and T_2_ ∼ 70 ms were achieved by adding 0.125 mM MnCl.

For validation purposes, the phantom’s B_1_ map was also determined with the actual flip‐angle (AFI) method.^12^ For AFI, the same imaging matrix was acquired as for the multi‐spin‐echo sequence. Furthermore, a nonselective excitation pulse with a flip angle of 45° was used with TR2/TR1 = 175 ms/35 ms, TE = 4.91 ms, a receiver bandwidth of 120 Hz/px, and RF phase‐difference increment of 129.3°.^35^ The AFI method took about 15 minutes to complete. The local flip angle α was estimated from the signal ratio r=S2/S1 and the TR ratio n=TR2/TR1=5 using $\alpha \approx arccos\left[\left(rn‐1\right)/\left(n‐r\right)\right]$.

Skull stripping for the figures was performed using the standard software package FSL (FMRIB Software Library v6.0, Oxford, United Kingdom).

### 2.6 Electrical properties reconstruction

Calculation of the relative permittivity $\varepsilon_{r}$ and the conductivity σ was based on the homogeneous Helmholtz equation,^24^, ^36^ yielding:

(7) $$\varepsilon_{r}=\;‐\frac{1}{\varepsilon_{0}\mu_{0}\omega^{2}}Re\left\{\frac{\nabla^{2}B_{1}^{+}}{B_{1}^{+}}\right\}=\frac{1}{\varepsilon_{0}\mu_{0}\omega^{2}}\left(‐\frac{\nabla^{2}\left|B_{1}^{+}\right|}{\left|B_{1}^{+}\right|}+{\left|\nabla \varphi^{+}\right|}^{2}\right)$$

(8) $$\sigma =\frac{1}{\mu_{0}\omega }Im\left\{\frac{\nabla^{2}B_{1}^{+}}{B_{1}^{+}}\right\}=\frac{1}{\mu_{0}\omega }\left(\nabla^{2}\varphi^{+}+2\frac{\nabla \left|B_{1}^{+}\right|\cdot \nabla \varphi^{+}}{\left|B_{1}^{+}\right|}\right).$$

In Equations 7 and 8, $\varepsilon_{0}$ is the vacuum permittivity; $\mu_{0}$ is the magnetic vacuum permeability; and ω is the Larmor frequency. The leading (first) terms in these expressions relate the permittivity and the conductivity to the curvature of the B_1_^+^ magnitude and its phase, respectively. The second terms can be seen as a correction, which is on the order of 10%–20% for typical EPs of human tissues at field strengths up to 3 T.^5^, ^36^ For the proposed CP method, $\left|B_{1}^{+}\right|$ is retrieved from the signal’s oscillatory behavior (e.g., signal matching with the dictionary), and $\varphi^{+}$ is estimated directly from the phase image of the first echo, using the transceive phase assumption.^23^ For AFI, the transceive phase from the CP sequence was taken for permittivity reconstruction.

The Laplacian and gradients were estimated using the coefficients of a locally fitted second‐order polynomial, restricted to voxels with magnitude values similar to the center point of the window (within 15%).^37^ This is a strategy to mitigate tissue‐boundary errors inherent to taking derivatives and assumes one EP value per tissue. The Laplace estimate was subsequently smoothed with a tissue boundary–preserving median filter.^25^, ^37^ For the phantom, the window size for the Laplace estimation was set to 7 × 7 × 7 voxels and to 21 × 21 × 21 voxels for subsequent median filtering. For the brain, the window size was 7 × 7 × 13 voxels for estimating the Laplacian and 15 × 15 × 21 voxels for the tissue‐preserving median filter. The magnitude image used for the EP reconstruction was a constructed T_2_‐weighted image that was generated based on the received T_2_ map and the magnitude image of the first echo in the measurement.

## 3 RESULTS

**FIGURE 2:**
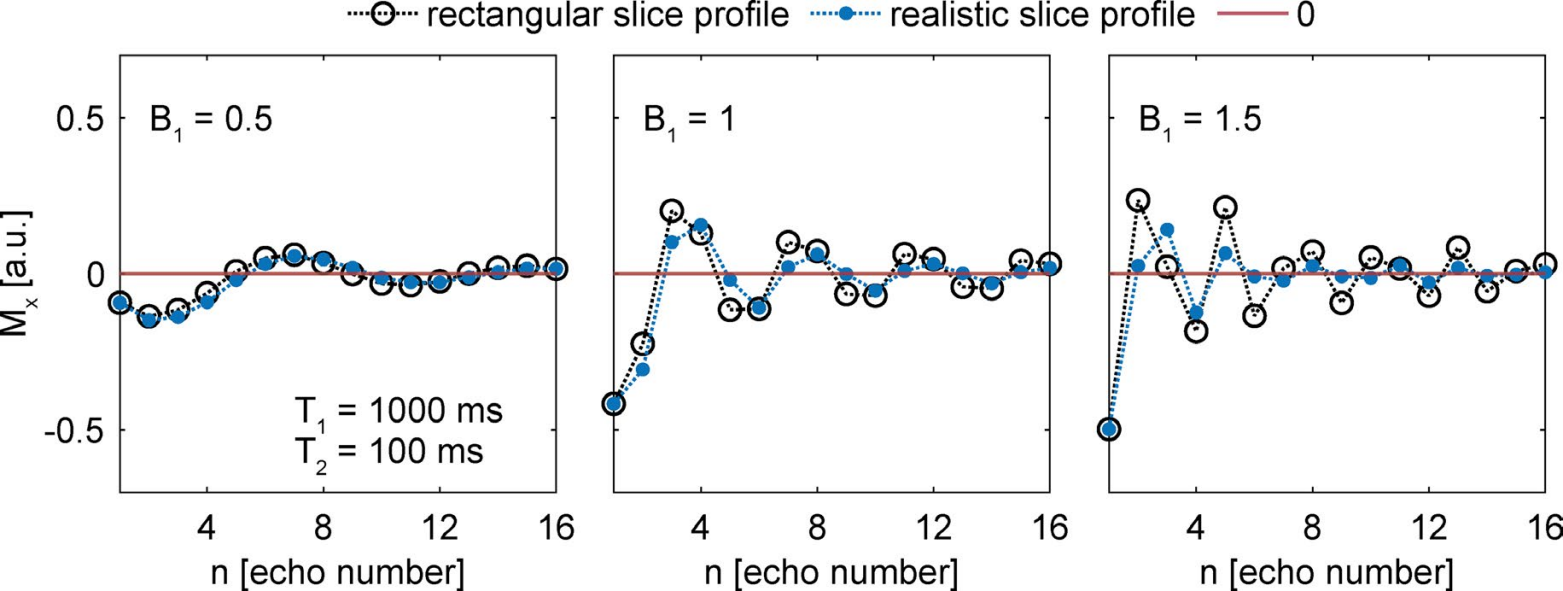
Simulations of CP echo amplitudes for an ideal slice profile (black open circles) and for a realistic slice profile (blue filled circles) for relative B_1_ values of 0.5, 1, and 1.5. The real slice profile was computed using the pulse shape generated from the sequence. The simulations were performed using the configuration model toolkit^29^

The CP echo signals only oscillates with an exact frequency of α/2π (see Equation 5) for ideal rectangular slice profiles. In practice, the slice profile is generally not rectangular, and the signal sums over a distribution of flip angles centered around the chosen flip angle α. As a result, the observed frequencies deviate from the ideal α/2π behavior (see Figure 2).

**FIGURE 3:**
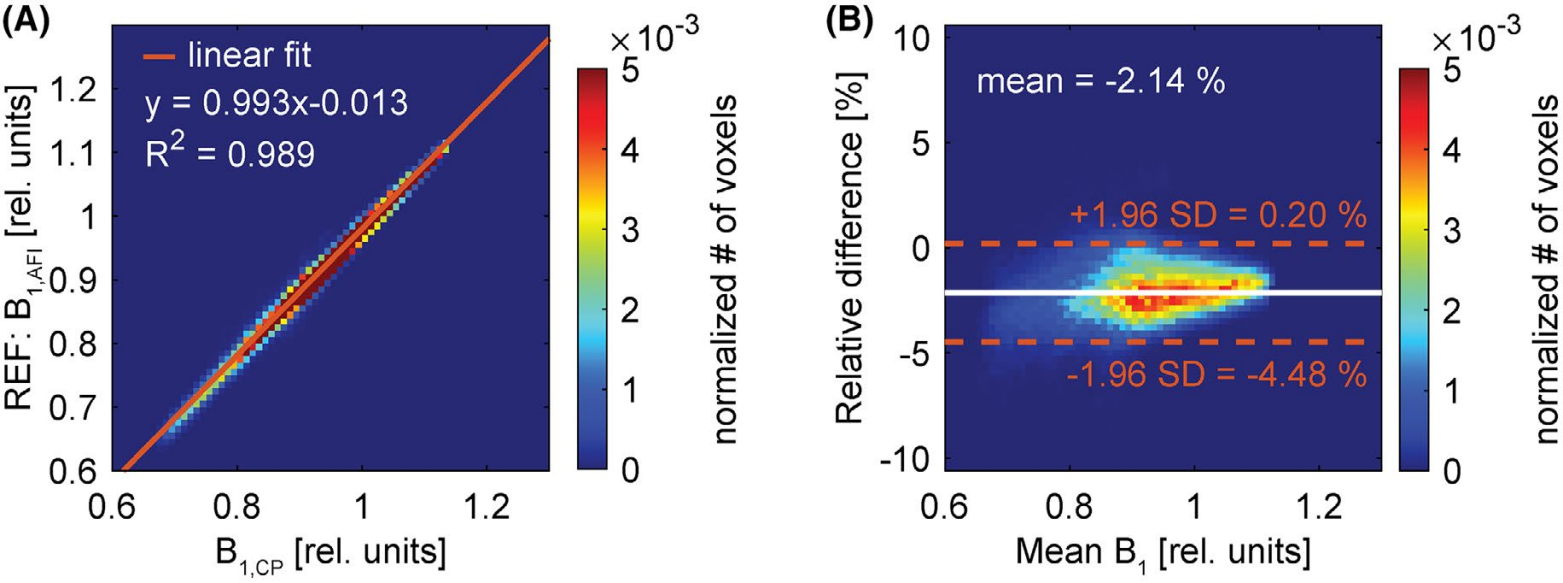
A, Binned scatter plot illustrating the correlation between the B_1,CP_ (x‐axis) and the B_1,AFI_ (y‐axis). The solid line is the result of a linear least‐square analysis. B, Corresponding Bland‐Altman plot of the relative difference of the two methods as a function of the voxel‐wise mean B_1_. The solid line corresponds to the mean relative difference in percentage. The dashed lines indicate the 95% confidence interval

Figure 3A shows a voxel‐wise comparison of the observed B_1_^+^ magnitude for both AFI and CP, and over the whole phantom in the form of a binned scatter plot. Using a linear regression analysis, a slope of 0.993 and an offset of −0.013 are found, indicating excellent correlation. In Figure 3B, the relative difference of the two methods is assessed in a Bland‐Altmann plot,^38^ where the relative difference is defined as (B_1,AFI_ − B_1,CP_)/(mean B_1_) for every voxel. The “mean B_1_” corresponds to the voxel‐wise average of the B_1,AFI_ and the B_1,CP_. The mean relative difference is calculated to be at −2.14% and is indicated in the figure together with the 95% confidence interval.

**FIGURE 4:**
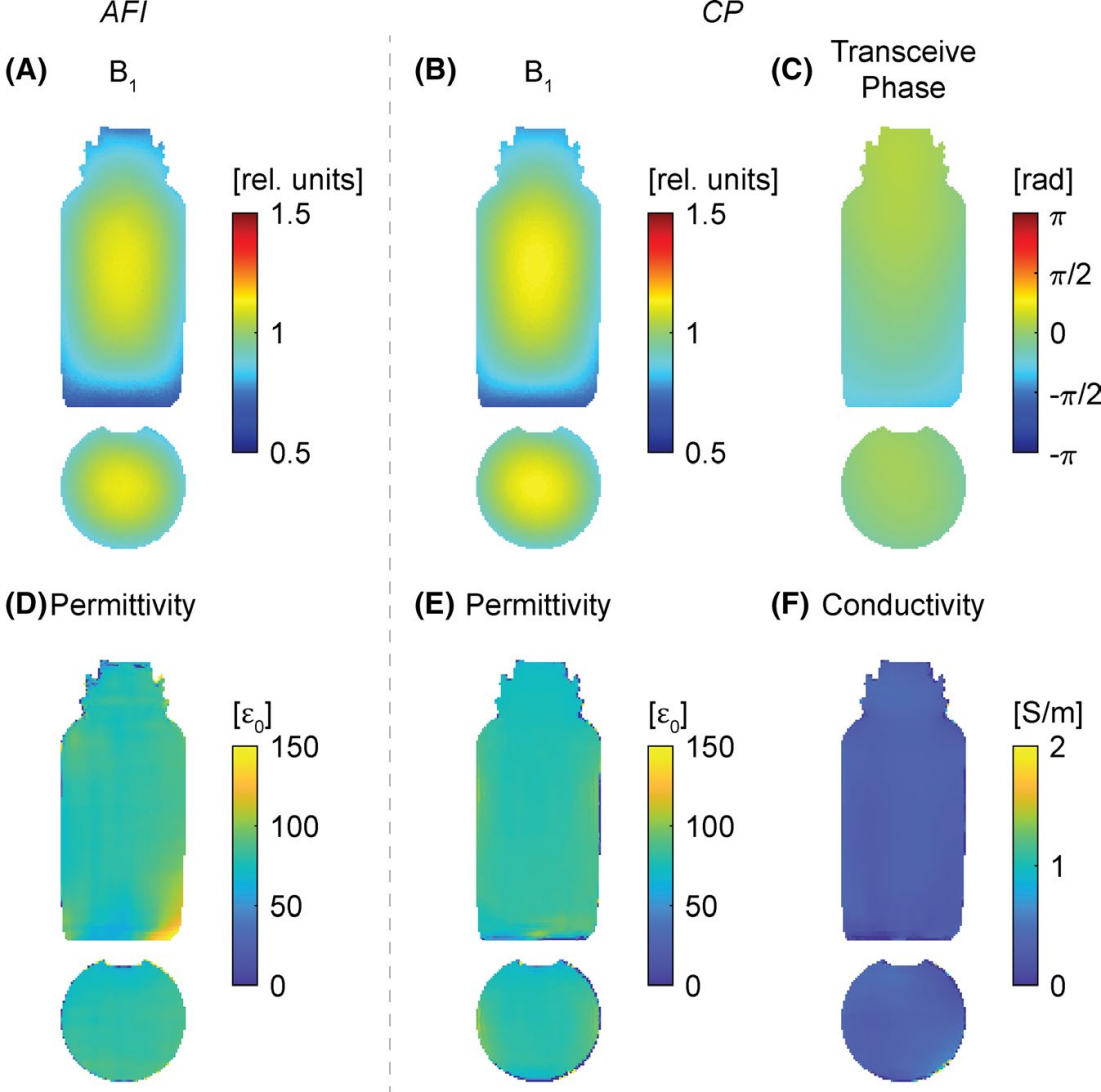
Exemplary coronal and axial slices of the saline phantom. A, B_1_ map obtained with actual flip‐angle imaging (AFI). B, B_1_ map obtained with the CP spin‐echoes method. C, Transceive phase obtained from the first echo acquisition. D, Reconstructed permittivity from the B_1_ map in (A). E, Reconstructed permittivity from the B_1_ map in (B). F, Reconstructed conductivity from the phase in (C)

Example B_1_ maps, shown in coronal and transversal orientation, are shown for the AFI scan in Figure 4A and for the CP scan in Figure 4B. The transceive phase images, retrieved from the CP scan, are shown in Figure 4C. The uncertainty of the transceive phase measurement was estimated to be about 0.005 rad. The corresponding permittivities based on the two B_1_ maps are shown in Figure 4D,E, respectively, and the CP‐based conductivity is shown in Figure 4F. For a large region in the center of the shown axial slice, a mean permittivity value of (80 ± 3) ε_0_ is found for AFI, whereas for the CP approach a mean permittivity value of (79 ± 3) ε_0_ and a mean conductivity value of (0.35 ± 0.04) S/m is observed. A comparison of the results using either the birdcage coil or the multichannel receive coil is shown in Supporting Information Figure S1. Overall, only marginal differences are observed between the phase information as retrieved by the manufacturer’s “adaptive combine” method and the phase information obtained using the birdcage coil.

**FIGURE 5:**
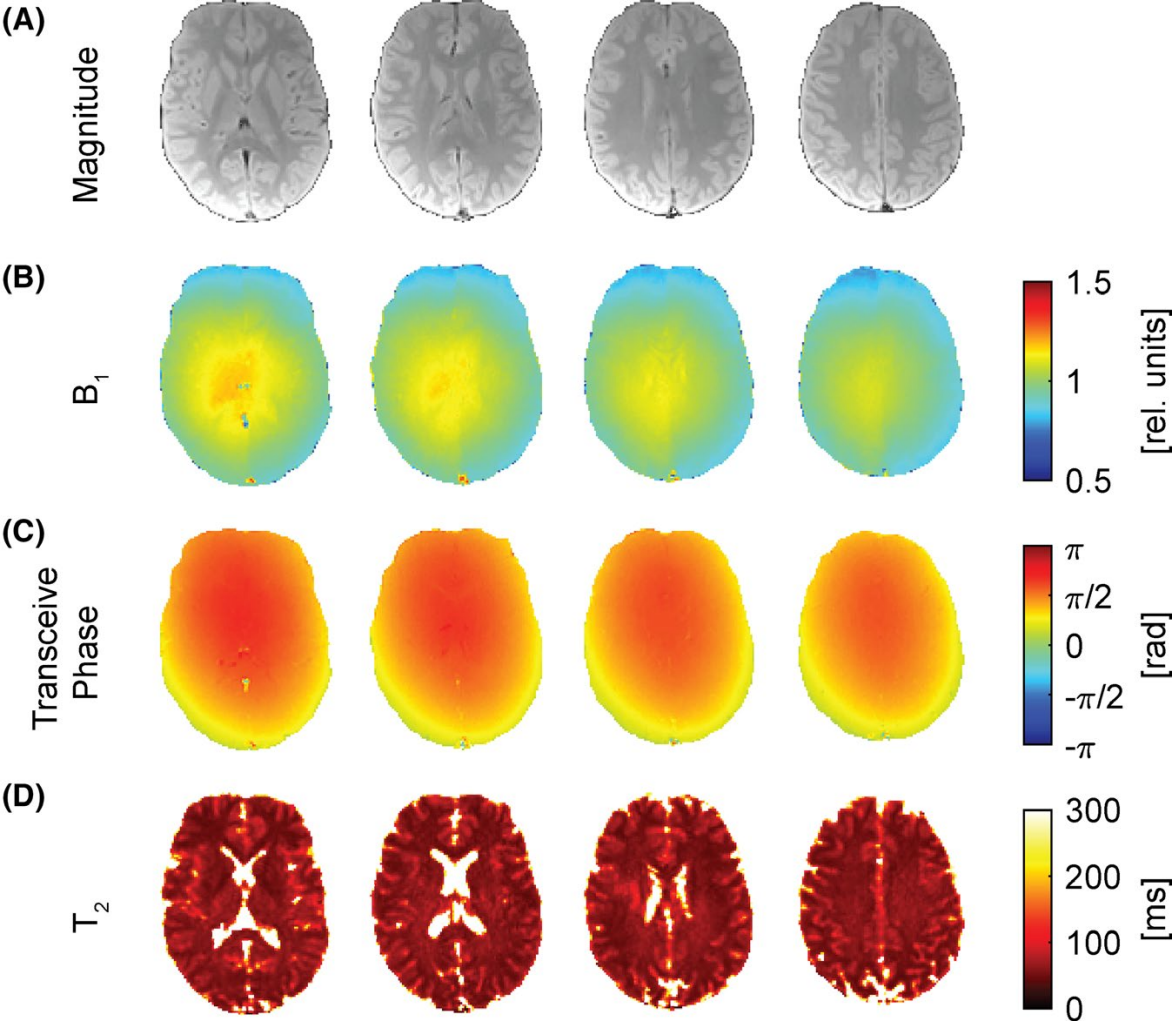
Exemplary axial slices of an in vivo brain. A, Magnitude brain images obtained from the first echo acquisition. B, B_1_ maps retrieved from the signal correlation with the dictionary. C, Transceive phase images obtained from the first echo acquisition. D, T_2_ maps retrieved from signal correlation with the dictionary

After validation in the phantom (Figure 4), the presented method was applied to in vivo brain scans. Figure 5 shows four exemplary axial slices of the brain of a volunteer and compiles the attainable maps. Figure 5A shows the anatomical magnitude images from the first echo for four exemplary axial slices. The contrast of the magnitude image depends considerably on the sequence parameters. The SNR was estimated to be about 80 for the brain scan. In Figure 5B, the B_1_ maps based on the presented method are shown. In the field maps, a distinct asymmetry between the left and right brain hemispheres is visually notable. Figure 5C compiles the (transceive) phase images obtained with the first echo of the same slices. The uncertainty of the phase measurement in the brain was found to be 0.013 rad. Finally, Figure 5D shows the quantitative T_2_ maps for the corresponding slices. The T_2_ maps are a direct result of the dictionary‐based reconstruction.

**FIGURE 6:**
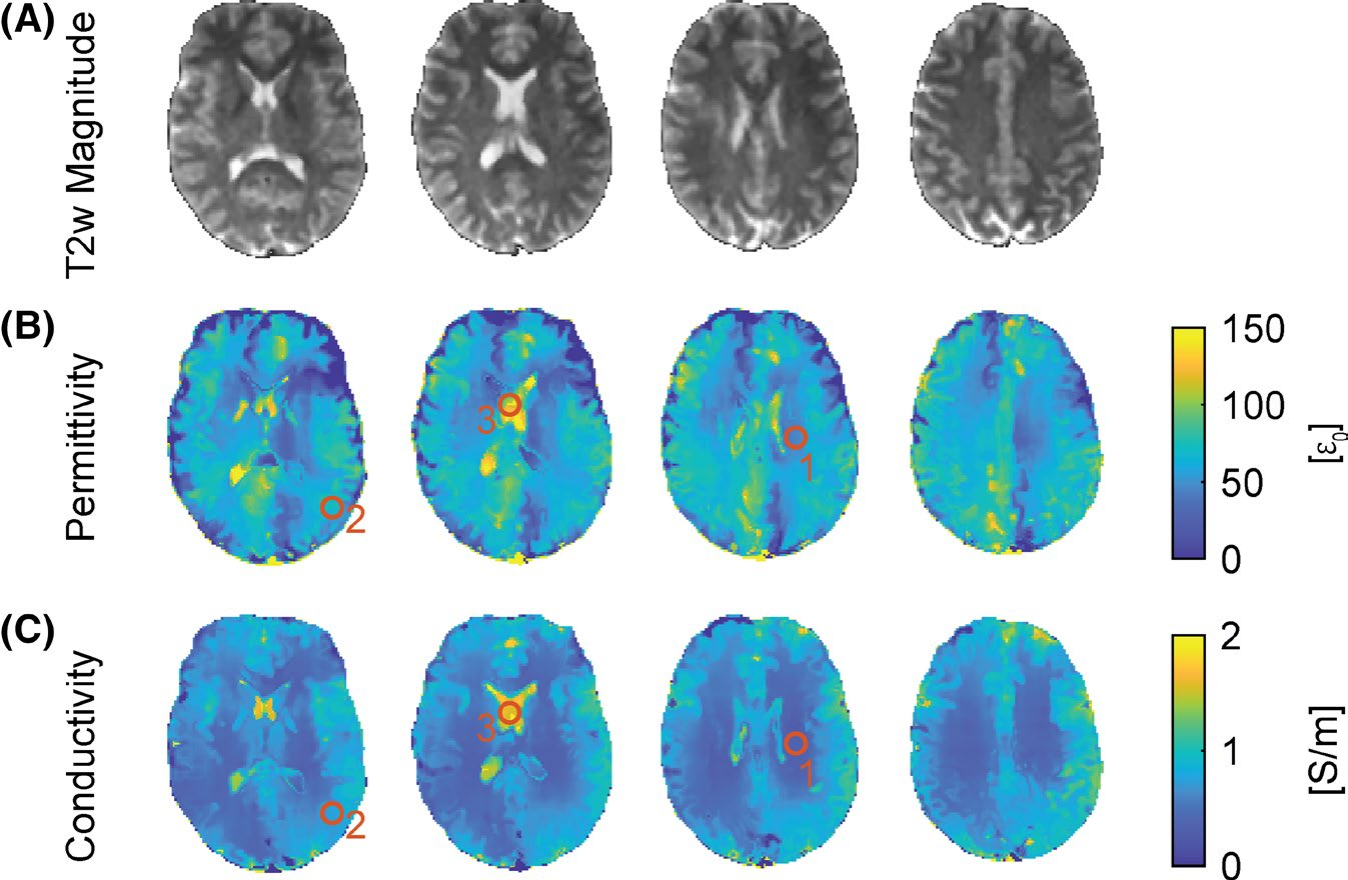
Same exemplary in vivo brain axial slices as shown in Figure 5. A, Generated T_2_‐weighted magnitude images for electrical properties reconstruction. B, Reconstructed permittivity. C, Reconstructed conductivity. The regions of interest (ROIs) indicate where the EP values are taken from with ROI 1 corresponding to white matter (WM), ROI 2 corresponding to gray matter (GM), and ROI 3 corresponding to CSF

For the same exemplary slices as in Figure 5, the results of the EP reconstruction are shown in Figure 6. Figure 6A shows the generated T_2_‐weigthed magnitude image on which the EP reconstruction is based. Corresponding maps of the electrical properties, permittivity, and conductivity are shown in Figure 6B,C. The asymmetry in the B_1_ map (see Figure 5B) of the brain presents an abrupt change in the field distribution, which can lead to biased estimation of the permittivity. For a selected region of interest (ROI) in white matter (WM), a permittivity of (50 ± 2) ε_0_ is found (ROI 1 in Figure 6B), whereas the reconstructed conductivity in the same location yields a value of (0.33 ± 0.01) S/m (ROI 1 in Figure 6C). For gray matter (GM), a permittivity of (69 ± 6) ε_0_ (ROI 2 in Figure 6B) and a conductivity of (0.75 ± 0.03) S/m (ROI 2 in Figure 6C) are obtained, whereas in CSF, a permittivity of (112 ± 14) ε_0_ (ROI 3 in Figure 6B) and a corresponding conductivity of (1.57 ± 0.04) S/m are found (ROI 3 in Figure 6C).

## 4 DISCUSSION

In this work, a novel method for simultaneous B_1_^+^ magnitude and phase estimation using a multi‐spin‐echo approach is presented. In particular, the focus lies on the CP multi‐spin‐echo sequence. The local flip angle can be retrieved from its oscillatory signal behavior, which is more pronounced in the CP sequence than in CPMG. To this end, we suggest using a dictionary for the analysis of the signal oscillations in conjunction with T_2_ quantification. A similar dictionary approach was implemented for improving CPMG‐based T_2_ quantification.^39^ Because the sequence is a spin‐echo sequence, the transceive phase is already contained in the measurement, more specifically in the first echo.

The flip angle–dependent frequency of the multi‐spin‐echo sequence can be analyzed in several ways. Looking for the prominent frequency of the Fourier transform of the time‐series appears like the most obvious approach but is not very robust and often inaccurate.^12^ The B_1_ mapping technique B1‐TRAP^12^ uses an analysis algorithm called “matrix pencil”,^40^ an approach that identifies a user‐defined number of frequency components with corresponding amplitude and damping. However, signals of 2D acquisitions are affected by pulse‐shape effects and may cause a bias in the matrix pencil frequency estimation, as only a limited number of components will be found. In contrast to directly assessing the frequency, signal analysis can also be performed using a dictionary, as suggested in this work. The dictionary can be set up with different MR signal simulation techniques such as using Bloch equations,^39^, ^41^ the extended phase graph formalism,^18^, ^19^ or as in this work, the configuration model toolkit.^29^ The advantage of a dictionary over direct frequency analysis is that relaxation and actual slice‐profile effects can be rather easily accounted for. The accuracy of the local flip‐angle estimation is then dependent primarily on the resolution of the dictionary. To accurately retrieve B_1_ information, the T_2_ decay must also be considered. A welcomed byproduct then is local T_2_ quantification that is obtained along with the B_1_ map. In theory, for refocusing pulse flip angles other than 180°, T_1_ relaxation also affects the signal. However, the influence of T_1_ is relatively small, and the dictionary is not sensitive enough to recover an accurate T_1_ map. In any case, ideally, because of the sensitivity to relaxation, the dictionary should be adjusted whenever the TR or especially TE is changed.

The use of a dictionary with a step size for the B_1_ values of 0.01 gives the B_1,CP_ visually a less noisy appearance than the B_1,AFI_, in which the B_1_ value can assume values on an almost continuous scale. Nonetheless, the comparison between the presented method and AFI showed good agreement of the two methods. The spin‐echo phase is often used as the benchmark transceive phase and thus does not require validation. In this case, as the transceive phase is directly contained in the measurement, its uncertainty is directly linked to the SNR of the acquisition.

Our results indicate that the transceive phase is properly estimated using the manufacturer’s “adaptive combine” algorithm for multichannel coil data (see Supporting Information Figure S1). The vendor‐specific software is, however, inaccessible for the typical user; thus, the exact implementation of the coil combination method is unknown. Therefore, generally, a possible bias in the transceive phase from the vendor’s coil combine algorithm cannot be ruled out. As the observed permittivity of (79 ± 3) ε_0_ and the conductivity of (0.35 ± 0.04) S/m values are in excellent agreement with the expected values (79 ε_0_, 0.34 S/m),^33^ the possible error is assumed to be small.

The obtained B_1_ maps of the brain scans indicate good translation of the method to in vivo conditions. As mentioned, the additional effort in generating the dictionary is compensated with the retrieval of additional quantitative T_2_ information. Therefore, the presented method is a multi‐parametric technique that allows the retrieval of several maps with a single sequence.

For brain tissues at 3 T, the expected permittivity values are 52 ε_0_ (WM), 73 ε_0_ (GM) and 84 ε_0_ (CSF), and the corresponding conductivity is expected to be about 0.34 S/m (WM), 0.59 S/m (GM), and 2.14 S/m (CSF).^42^, ^43^ The obtained values for the conductivity ((0.33 ± 0.01) S/m (WM), (0.75 ± 0.03) S/m (GM), and (1.57 ± 0.04) S/m (CSF)) agree with expectations for brain tissues. The deviations might be attributed to large‐scale filtering and partial‐volume effects due to unproper separation of tissues. Except for CSF, also the found permittivity values ((50 ± 2) ε_0_ (WM), (69 ± 6 ε_0_) (GM), and (112 ± 14) ε_0_ (CSF)) are close to expectations. Overall, however, the permittivity map shows some inhomogeneity in brain tissues. It has already been reported that permittivity mapping at 3 T based on the Helmholtz equation is rather challenging in clinical acceptable scan times.^44^

Another source of error might arise from the use of a magnitude image to mitigate boundary errors in the reconstruction of the Laplacian.^37^ For this geometric constraint, any high‐contrast image can be used. The contrast of the first‐echo image of the CP scan is dominated by the proton density, whereas the T_2_ map from the dictionary mainly separates fluids from tissue. Thus, in this work, we used both the proton density and the quantitative T_2_ map to generate a T_2_‐weighted image that offered the desired level of tissue contrast.

Other factors might also play a role in compromising the accuracy of the electrical properties. Especially the B_1_ asymmetry introduces an erroneous region in the permittivity across the brain (see Figure 6). The source of the asymmetry in the excitation across the brain is not exactly known but is seen throughout other works when an unfiltered B_1_ map of the brain is shown.^45^ The strong variation in the B_1_ map can be smoothened out with appropriate postprocessing at the cost of resolution. However, optimizing the reconstruction of the EPs is beyond the scope of this work.

## 5 CONCLUSIONS

We showed that conventional CP multi‐spin‐echo sequences can be exploited for B_1_ mapping. In conjunction with the acquired spin‐echo phase, all of the means to perform EPT for both permittivity and conductivity are provided. The presented method thus offers a good estimate of the complex‐valued B_1_^+^. As the signal of the echo train is dependent primarily on the local flip angle and transverse relaxation, the analysis with the dictionary also provides a quantitative T_2_ map. EPT was performed with the complex EPT reconstruction technique based on the homogeneous Helmholtz equation and showed good agreement with expectations for the phantom. In vivo, the obtained EPs showed some variation from expectations but generally also agreed well with what has been reported and yielded qualitatively good EP maps. In summary, the use of CP multi‐spin‐echo approaches might boost efficiency and could render EPT clinically relevant.

## Supporting information


**FIGURE S1** Example coronal and axial slices of the saline water phantom using the proposed CP sequence. A, Transceive phase measured with a birdcage coil. B, Transceive phase using the manufacturers “adaptive combine” method for the data from the 20‐channel head and neck coil. A global offset was added to the head and neck coil phase measurement for comparison purposes. The measurements were carried out as described in the methods section except that parallel imaging is not available for the birdcage coil. C, Conductivity reconstruction based on the birdcage coil data. D, Conductivity reconstruction based on 20‐channel head and neck coil data. The conductivity was reconstructed as described in the methods section using the simultaneously obtained B_1_ map for the “full” complex electrical properties tomography (EPT) method. E, Histogram showing the distribution of the obtained conductivity values in the range between 0 S/m and 0.8 S/m for both coils. In this range, the birdcage and the head and neck coil yield a mean conductivity value of (0.37 ± 0.09) S/m and (0.36 ± 0.10) S/m, respectively.
