# Supplementary material for: Complex B1 + mapping with Carr‐Purcell spin echoes and its application to electrical properties tomography
Source: Magn Reson Med. 2021 Nov 9;87(3):1250–60. doi: 10.1002/mrm.29020 (PMC9298742; doi:10.1002/mrm.29020)
Supplement: Supplementary file 1 — FIGURE S1 Example coronal and axial slices of the saline water phantom using the proposed CP sequence. A, Transceive phase measured with a birdcage coil. B, Transceive phase using the manufacturers “adaptive combine” method for the data from the 20‐channel head and neck coil. A global offset was added to the head and neck coil phase measurement for comparison purposes. The measurements were carried out as described in the methods section except that parallel imaging is not available for the birdcage coil. C, Conductivity reconstruction based on the birdcage coil data. D, Conductivity reconstruction based on 20‐channel head and neck coil data. The conductivity was reconstructed as described in the methods section using the simultaneously obtained B1 map for the “full” complex electrical properties tomography (EPT) method. E, Histogram showing the distribution of the obtained conductivity values in the range between 0 S/m and 0.8 S/m for both coils. In this range, the birdcage and the head and neck coil yield a mean conductivity value of (0.37 ± 0.09) S/m and (0.36 ± 0.10) S/m, respectively. [file MRM-87-1250-s001.pdf]

Complex B_1^+ Mapping with Carr-Purcell Spin Echoes and its Application to Electrical Properties Tomography

Supporting Information

Santhosh Iyyakkunnel[1,2], Matthias Weigel[1,2,3,4], Carl Ganter[5] and Oliver Bieri[1,2]

[1]*Division of Radiological Physics, Department of Radiology, University Hospital Basel, Basel, Switzerland*

[2]*Department of Biomedical Engineering, University of Basel, Basel, Switzerland*

[3] *Translational Imaging in Neurology (ThINk) Basel, Department of Biomedical Engineering, Faculty of Medicine, University Hospital Basel and University of Basel, Basel, Switzerland*

[4] *Neurologic Clinic and Policlinic, MS Center and Research Center for Clinical Neuroimmunology and Neuroscience Basel (RC2NB), University Hospital Basel and University of Basel, Basel, Switzerland*

[5]*Department of Radiology, Klinikum rechts der Isar, Technical University of Munich, Munich, Germany*

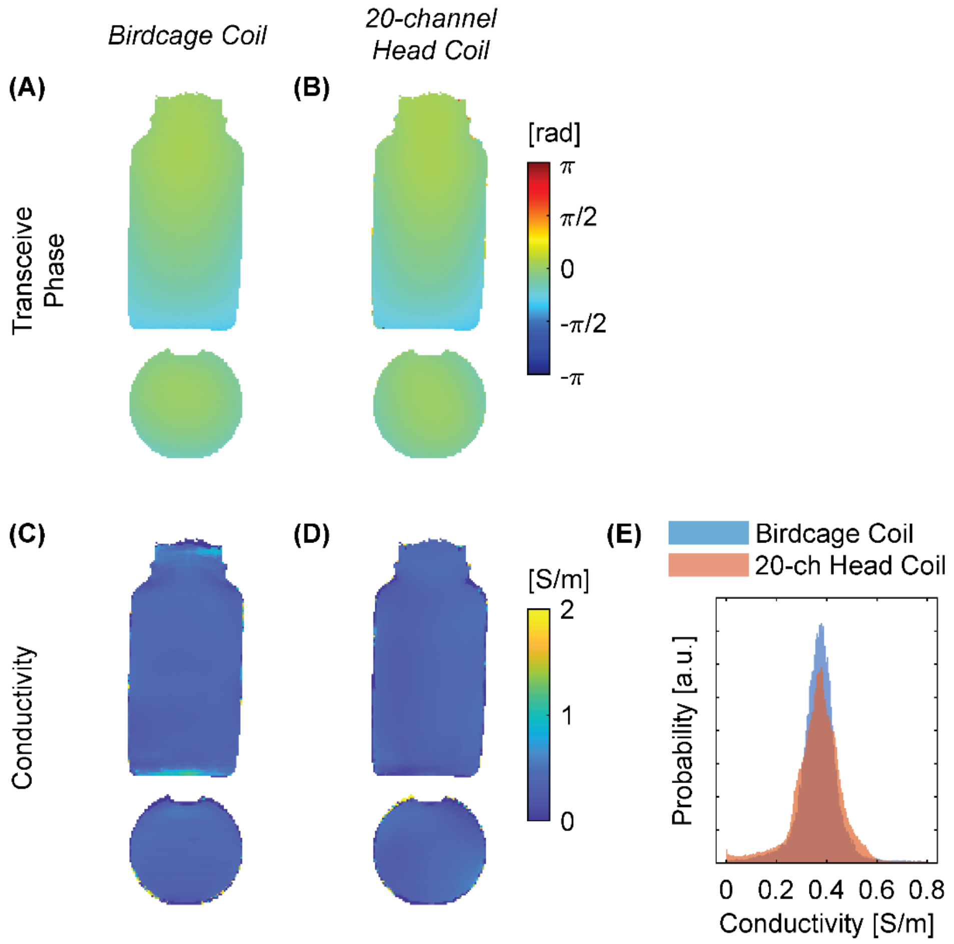

Figure S1 Example coronal and axial slices of the saline water phantom using the proposed CP sequence. A, Transceive phase measured with a birdcage coil. B, Transceive phase using the manufacturers "adaptive combine" method for the data from the 20-channel head and neck coil. A global offset was added to the head and neck coil phase measurement for comparison purposes. The measurements were carried out as described in the methods section except that parallel imaging is not available for the birdcage coil. C, Conductivity reconstruction based on the birdcage coil data. D, Conductivity reconstruction based on 20-channel head and neck coil data. The conductivity was reconstructed as described in the methods section using the simultaneously obtained B_1 map for the "full" complex electrical properties tomography (EPT) method. E, Histogram showing the distribution of the obtained conductivity values in the range between 0 S/m and 0.8 S/m for both coils. In this range, the birdcage and the head and neck coil yield a mean conductivity value of (0.37 ± 0.09) S/m and (0.36 ± 0.10) S/m, respectively.
